# Three-dimensional comparison between the effects of mandibular advancement device and maxillomandibular advancement surgery on upper airway

**DOI:** 10.1186/s12903-023-03125-5

**Published:** 2023-06-30

**Authors:** Marcela Gurgel, Lucia Cevidanes, Fabio Costa, Rowdley Pereira, Paulo Cunali, Lia Bittencourt, Antonio Ruellas, Joao Gonçalves, Jonas Bianchi, Cauby Chaves

**Affiliations:** 1 Department of Dental Clinic, School of Dentistry, Federal University of Ceará, Fortaleza, 1273 Monsenhor Furtado St, CE Brazil; 2 Department of Orthodontics and Pediatric Dentistry, School of Dentistry, University of Michigan, Ann Arbor, United States of America; 3 Department of Pneumology, Division of Sleep Medicine and Biology, Federal University of Sao Paulo, Sao Paulo, Brazil; 4 Department of Orthodontics and Pediatric Dentistry, School of Dentistry, Federal University of Rio de Janeiro, Rio de Janeiro, Brazil; 5 Department of Pediatric Dentistry, School of Dentistry, Sao Paulo State University (Unesp), Araraquara, Brazil; 6 Department of Orthodontics, University of the Pacific, Arthur A. Dugoni School of Dentistry, San Francisco, CA United States of America

**Keywords:** Cone-Beam Computed Tomography (CBCT), Three-dimensional assessment, Upper airway, Obstructive sleep apnea, Maxillomandibular advancement, Mandibular advancement device

## Abstract

**Background:**

The efficacy of mandibular advancement devices (MAD) and maxillomandibular advancement (MMA) in improving upper airway (UA) patency has been described as being comparable to continuous positive airway pressure (CPAP) outcomes. However, no previous study has compared MAD and MMA treatment outcomes for the upper airway enlargement. This study aimed to evaluate three-dimensionally the UA changes and mandibular rotation in patients after MAD compared to MMA.

**Methods:**

The sample consisted of 17 patients with treated with MAD and 17 patients treated with MMA matched by weight, height, body mass index. Cone-beam computed tomography from before and after both treatments were used to measure total UA, superior/inferior oropharynx volume and surface area; and mandibular rotation.

**Results:**

Both groups showed a significant increase in the superior oropharynx volume after the treatments (p = 0.003) and the MMA group showed greater increase (p = 0.010). No statistical difference was identified in the MAD group considering the inferior volume, while the MMA group showed a significantly gain (p = 0.010) and greater volume (p = 0.024). Both groups showed anterior mandibular displacement. However, the mandibular rotation were statistically different between the groups (p < 0.001). While the MAD group showed a clockwise rotation pattern (-3.97 ± 1.07 and − 4.08 ± 1.30), the MMA group demonstrated a counterclockwise (2.40 ± 3.43 and 3.41 ± 2.79). In the MAD group, the mandibular linear anterior displacement was correlated with superior [p = 0.002 (r=-0.697)] and inferior [p = 0.004 (r = 0.658)] oropharynx volume, suggesting that greater amounts of mandibular advancement are correlated to a decrease in the superior oropharynx and an increase in the inferior oropharynx. In the MMA group, the superior oropharynx volume was correlated to mandibular anteroposterior [p = 0.029 (r=-0.530)] and vertical displacement [p = 0.047 (r = 0.488)], indicating greater amounts of mandibular advancement may lead to a lowest gain in the superior oropharynx volume, while a great mandibular superior displacement is correlated with improvements in this region.

**Conclusions:**

The MAD therapy led to a clockwise mandibular rotation, increasing the dimensions of the superior oropharynx; while a counterclockwise rotation with greater increases in all UA regions were showed in the MMA treatment.

**Supplementary Information:**

The online version contains supplementary material available at 10.1186/s12903-023-03125-5.

## Introduction

Upper airway (UA) anatomy and patency are related to different sleeping breath disorders that plays an important role in the development of symptoms, such as anxiety and depression, and sleep patter cardiovascular changes are associated with a significant increase in mortality risk [1–3]. Several therapies had been proposed for UA patency maintenance. Continuous positive airway pressure (CPAP) is often considered the gold standard treatment for obstructive sleep apnea. However, recent expert recommendations suggest that mandibular advancement devices (MADs) may be just as effective as CPAP for mild to moderate cases of sleep apnea[4]. Moreover, when there is no acceptance or compliance to CPAP, MAD therapy or orthognathic surgery may be required as treatment alternatives [5–7].

The MAD therapy mechanism of action is based on gradual advancement of the mandible and distention of the UA tissues with the use of a removable intraoral device [1, 8]. Regarding to surgical procedures, the maxillomandibular advancement (MMA) is currently the first choice skeletal surgery for airway enlargement in adult patients [9]. In MMA, the maxillomandibular complex and pharyngeal airway muscles are repositioned to simultaneously increase pharyngeal soft tissue tension [10].

The efficacy of MAD and MMA in improving UA patency has been described as being comparable to CPAP outcomes [11]. However, no previous study has compared MAD and MMA treatment outcomes for the upper airway enlargement. Also, MADs are widely spread and the working mechanisms and effects are not completely understood. We hypothesize that there are different patterns of mandibular movements, as well as different effects in volume and area of UA when comparing MAD and MMA mandibular advancements. In this context, the aim of this study is to evaluate in three-dimensions (3D) the UA changes and mandibular rotation of patients after MAD and MMA treatment.

## Materials and methods

### Study design

This is a retrospective cohort study that compares CBCT scans taken before and after treatment of two groups: patients treated with MAD and patients treated with MMA, matched by weight, height, body mass index (BMI), as well clinical indication for mandibular advancement and increase of the airway. All patients had CBCTs taken before treatment (T0) and 6 months to one year after MAD or MMA (T1). The inclusion criteria were available CBCT scans from adults (older than 19 years) at both T0 and T1 time points with good image quality for accurate assessment of the areas of interest; matching height and weight in the study groups. Subjects who did not have matching height and weight were excluded.

### Ethical considerations

This study was approved by the Research Ethics Committee of the Federal University of São Paulo – Brazil (number 0301/10) (MAD treatment) and by Research Ethics Committee of São Paulo State University (number 3.717.097) (MMA treatment). All volunteers signed the informed consent form (ICF) and had their privacy rights assured.

### Sample size

Sample size calculation was performed using the findings of Consellu et al. [12]. Using measures of the total airway volume (+ 1261.6 ± 1476.2 mm³), we estimated at least 16 patients in two time points in the present study in order to obtain a sample that represents with 90% power and 95% confidence the alternative hypothesis of this work (paired t-test).

### MAD treatment

**Fig. 1:**
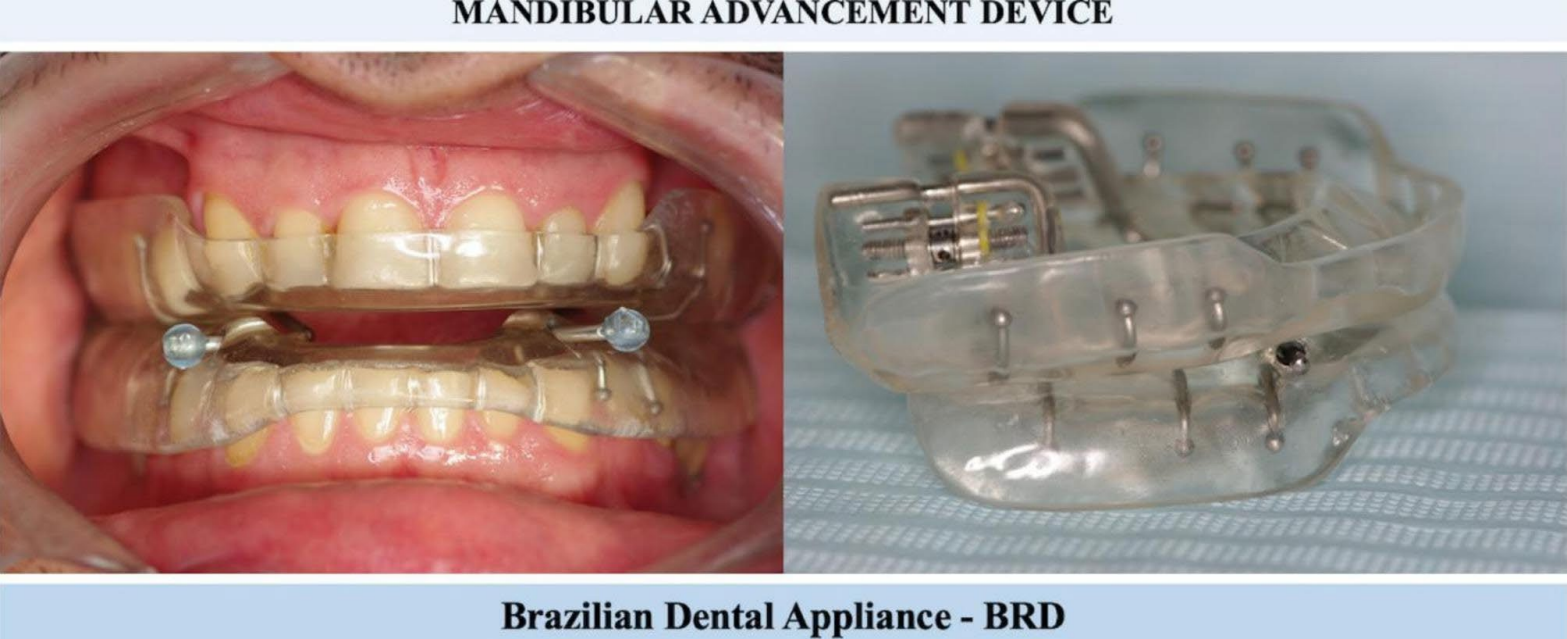
Mandibular advancement device used in the MAD study group

The MAD group patients were diagnosed with Obstructive Sleep Apnea by a physician specialist in sleep medicine after clinical and polysomnographic (PSG) exams. The MAD treatment was performed using the Brazilian dental appliance (BRD) [6], which is an individualized MAD that allows gradual mandibular advances, increasing the UA patency (Fig. 1). The BRD was developed through a partnership between the Federal University of Ceara and the Federal University of Sao Paulo in Brazil. The initial advancement was 50% of the total mandibular maximum protrusion capacity from each patient individually. The mandibular advancement was made gradually with 1 mm per week until achieving therapeutic protrusion and the record of the mandibular protrusion was obtained with the George’s Gauge. The OSA treatment success in this study considered a ≥ 50% AHI reduction from baseline or at least an AHI of < 10 events/hour [13].

### MMA treatment

In the MMA group, the patients showed skeletal maxillo-mandibular deficiency associated with a narrow UA and were diagnosed by a Dentist specialized in orthognathic surgery. The MMA group did not perform PSG exams or any other diagnostic tools to confirm the presence of sleep disorders. The patients were treated with two-jaw surgery to allow maxillomandibular advancement performed by the same experienced surgeon. Thus, osteotomies were performed in maxilla, being stabilized with 4 bone plates associated to 2 mm diameter screws and bone grafting whether necessary. The mandibular advancement surgery was performed by bilateral mandibular sagittal split osteotomies. In order to stabilize the mandibular repositioning, 1 bone plate was allocated in the posterior body region and 2–3 in the bicortical portion and 2 mm diameter screws were placed in the ascending ramus on each side [14].

### Variables

The demographic variables included the anthropometric characteristics: sex, age, weight, height, and BMI. Three-dimensional image analysis variables included UA volume and area, and mandibular linear and angular measurements.

#### CBCT acquisition

CBCT images from both groups were performed at a private dental radiological clinics (Sao Paulo, Brazil) using the i-CAT® scanner (Imaging Sciences International, Hatfield, PA), configured with 120Kvp, 3-8mA and 0.4 mm voxel size and field of view (FOV) of 23 cm x17 cm, allowing the total vertical head framing [15, 16]. During the image acquisition, all patients were in an upright posture, awake, in natural head position (Camper’s horizontal plane parallel to the ground) and were instructed to gaze at a stationary point on the wall. CBCT scans were taken in maximum intercuspal position [15, 17, 18]. All patients were instructed to not move, swallow or take deep breaths during the exam in order to avoid changes in the UA volume [19]. The images were stored in DICOM files (digital imaging and communications in medicine).

### Image processing and measurements

**Fig. 2:**
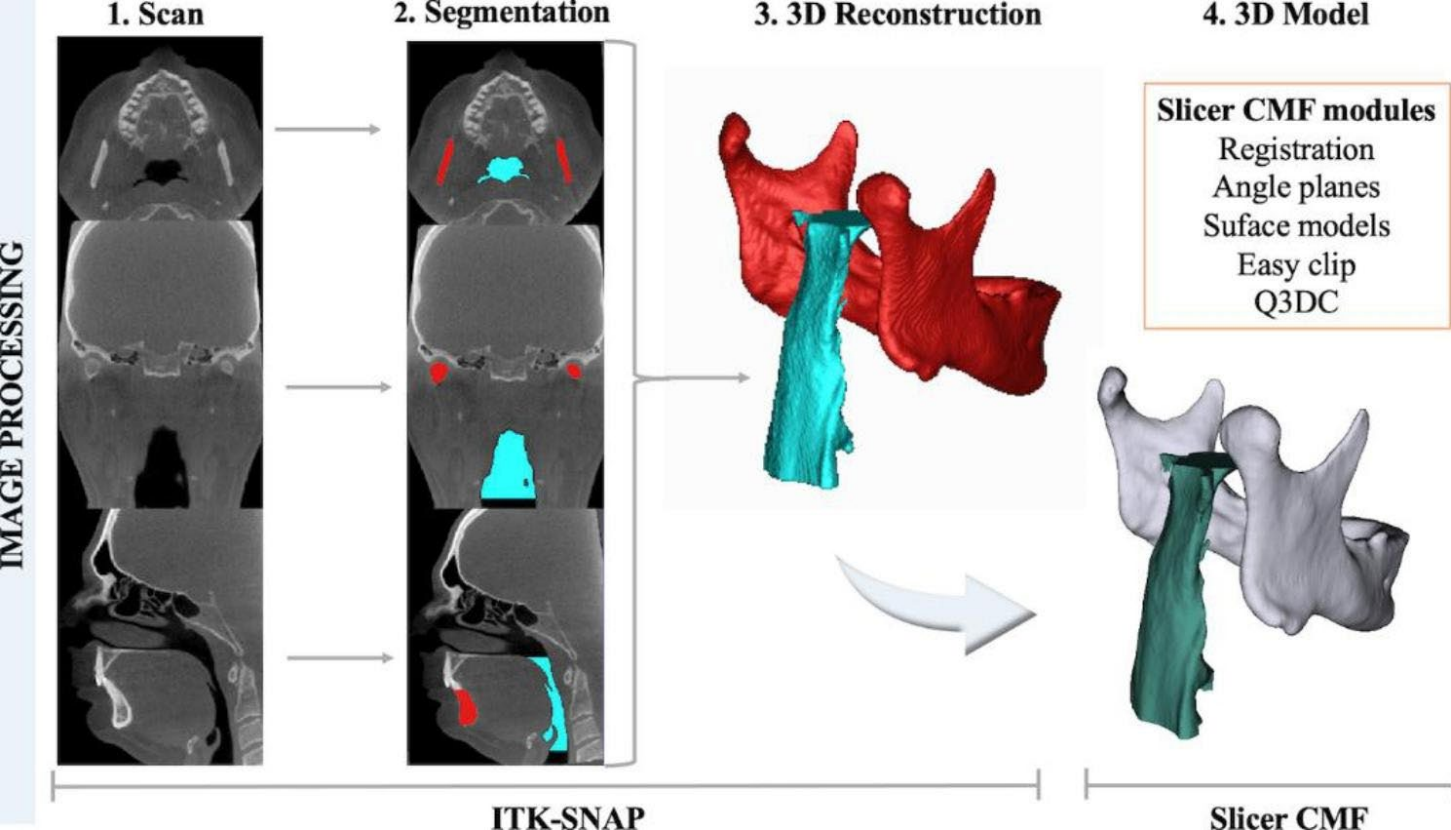
Image processing

**Fig. 3:**
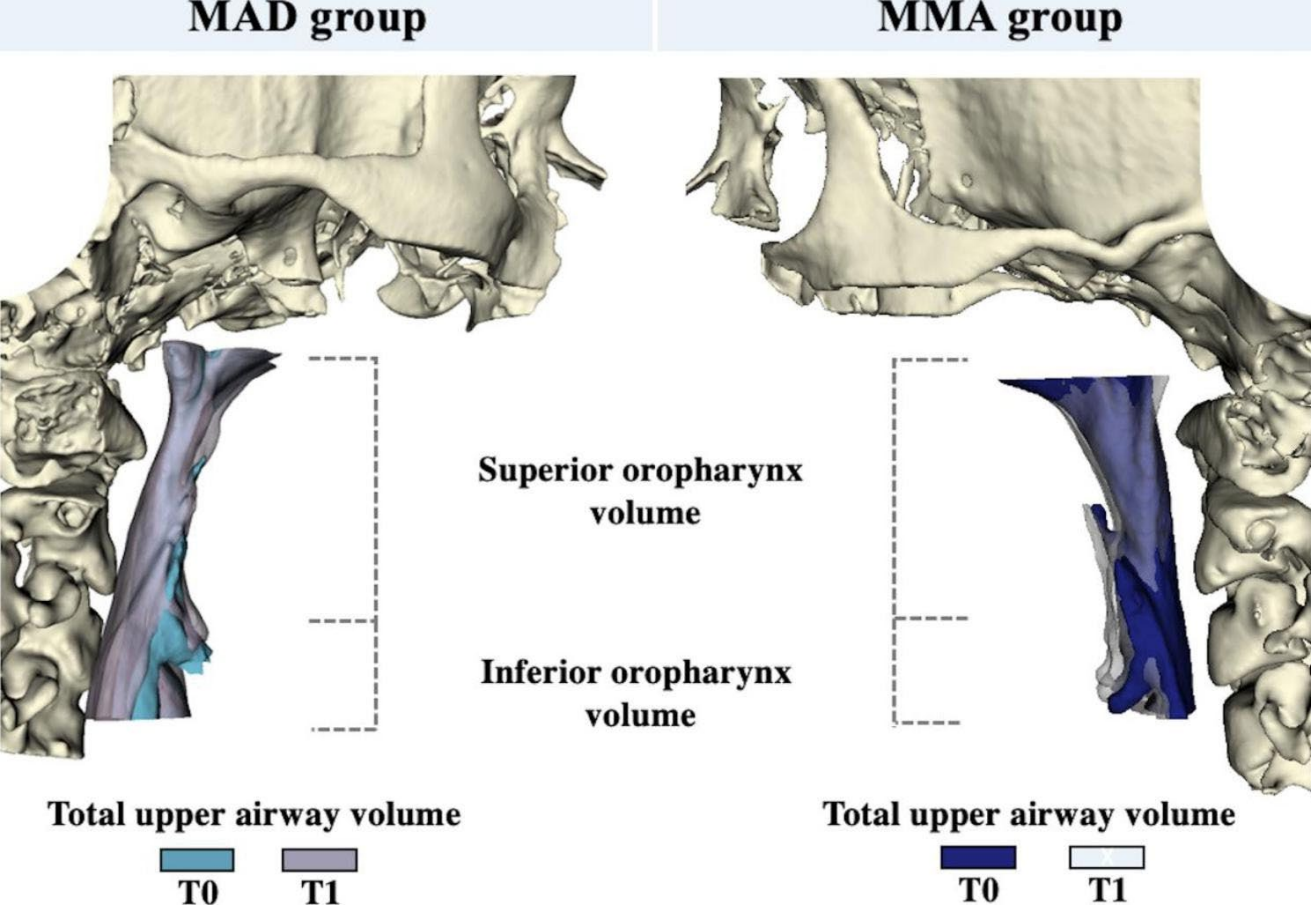
Total upper airway, superior and inferior oropharynx volume before (T0) and after (T1) Mandibular advancement device (MAD) and maxillomandibular advancement (MMA) treatments

**Fig. 4:**
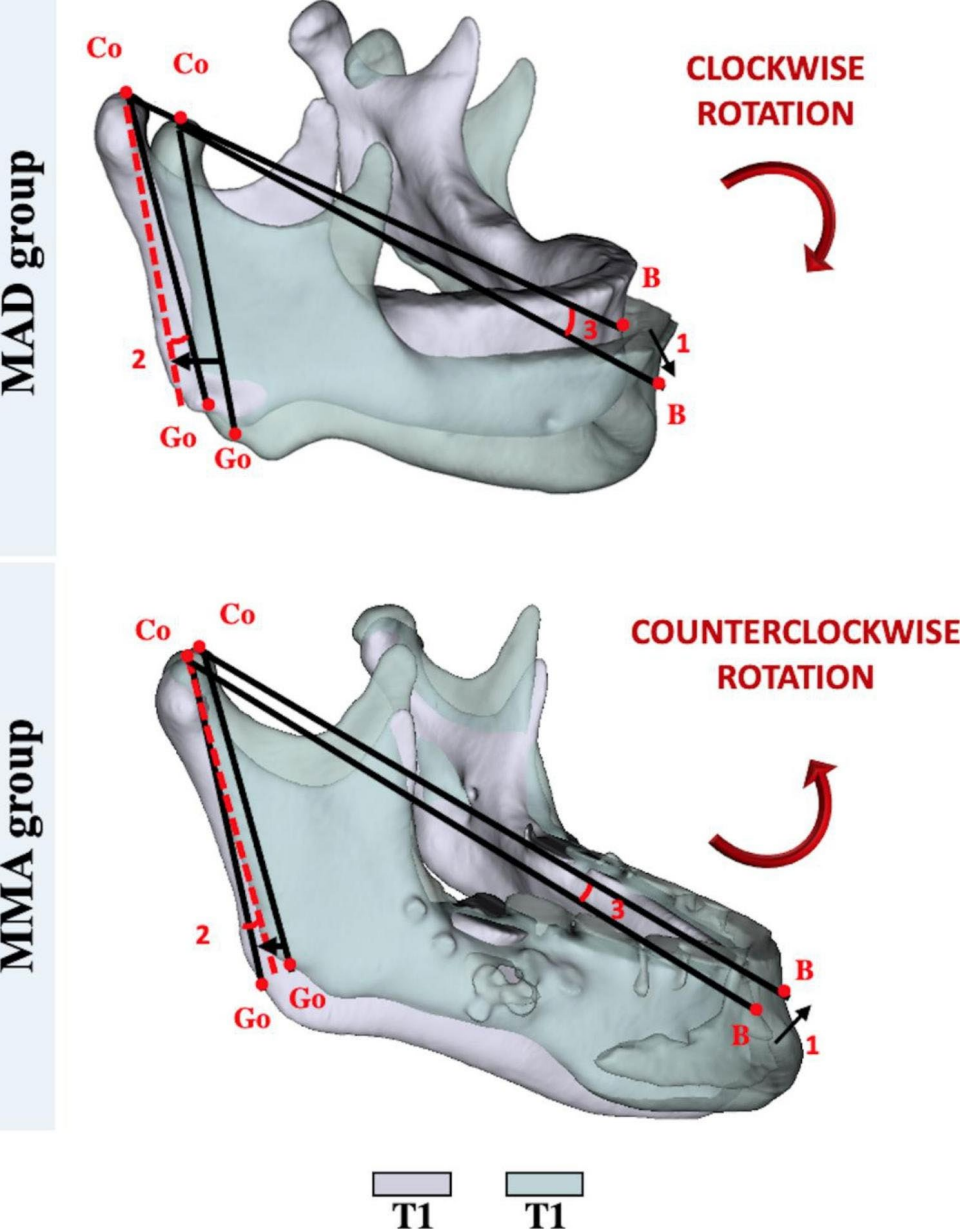
Mandibular measurements. (1) Mandibular linear displacement; (2) Mandibular ramus angular rotation; (3) Mandibular anterior angular rotation. Co = Condylion. Go = Gonion. B = B point. Maxillomandibular advancement (MMA).

Open-source imaging platforms were used to process all CBCT data at T0 and T1. ITK-SNAP 2.4 software (https://www.itksnap.org) was used to convert the DICOM in NIfTI files and obtain the segmentation required for image analysis. Orientation, registration and digital surface model creation of patients’ scans/segmentations, as well as all linear, angular and volumetric/area measurements, were performed using 3D Slicer software 4.11 (www.slicer.org) (Fig. 2). The digital models were moved by orienting their Frankfurt horizontal, midsagittal and transporionic planes to match the axial, sagittal and coronal planes, respectively, at a standard coordinate system from Slicer software, in order to apply the 3D head orientation for all T0 scans. Voxel based cranial registration was performed between the manually approximated T1 scan approximation to the oriented T0 scan [20]. Subsequently to the scan orientation and registration, semi-automated segmentations were performed in T0 and T1 CBCTs with ITK-SNAP 2.4 software (https://www.itksnap.org) and 3D digital models were created with the tool “model maker” present in the 3D Slicer software 4.11 (www.slicer.org). The digital models oriented (T0) and registered (T1) were uploaded simultaneously to the 3D Slicer software 4.11 (www.slicer.org), allowing accurate superimposition and evaluation of the UA and mandible rotation with the therapies (Figs. 2, 3 and 4).

To perform all measurements, a list of 3D landmarks was used for mandible and UA (Supplementary Table S1). All linear, angular, area, and volumetric dimensions were obtained, respectively, in millimeters (mm), degrees (°), squared millimeters (mm^2^) and cubic millimeters (mm^3^).

#### Upper airway measurements

In order to identify UA volume and area in T0 and T1 from the two groups, UA was delimited in superior oropharynx and inferior oropharynx and 3 measurements were performed (Fig. 3):

- Total upper airway volume/surface area: From Ba-PNS to C4S level (parallel to Ba-PNS).
- Superior oropharynx volume/surface area: From Ba-PNS to C2I level (parallel to Ba-PNS).
- Inferior oropharynx volume/surface area: From C2I to C4S (parallel to Ba-PNS).

#### Mandibular measurements

Three mandibular measurements were performed in both groups (Fig. 4):

- Mandibular linear displacement: Anteroposterior and vertical dimension between B point from T0 and T1 images.
- Mandibular ramus angular rotation: Right and left angle obtained by intersecting the line formed between Co-Go from T0 and the line formed between Co-Go from T1, considering the angle of pitch in the 3D space.
- Mandibular anterior angular rotation: Right and left angle obtained by intersecting the line formed between Co-B from T0 and the line formed between Co-B from T1, considering T0 and the line formed between Co-Go from T1, considering the angle of pitch in the 3D space.

### Study error

Intraexaminer reliability was performed blindly by one experienced examiner (MLG), repeating the 3D measurements with an interval of 15 days between the measurements in order to avoid potential sources of bias. The data were exported to Microsoft Excel spreadsheets (Microsoft Corporation, Redmond, WA) and analyzed using the Statistical Package for the Social Sciences (SPSS®) version 20.0 for Windows (IBM Corporation, Sommers, NY). Intraclass correlation coefficient (ICC) to evaluate systematic errors regarding numerical data and Dahlberg’s formula for assessing casual errors of measurements were performed.

### Statistical methods

The data were stored in Microsoft Excel and exported to the SPSS® software version 20.0 for Windows, in which the analyzes were performed adopting 95% confidence. Mean and standard deviation were calculated from all measures. Kolmogorov-Smirnov normality test was also applied for all the variables. Moreover, Student’s t test was made in order to compare MAD and MMA groups, as well as T0 and T1 CBCTs. Left and right sides were submitted to paired t test (parametric data). The multifactorial ANOVA test was used in all variables in order to adjust age factor and group factor. The variables correlations were analyzed by Pearson correlation.

## Results

### Study error

The intraexaminer repeatability of angular and linear measurements showed excellent correlation coefficients with ICC ranging from 0.920 to 0.999). The volume and area measurements also showed adequate ICC, ranging from 0.993 to 0.997. The Dahlberg’s coefficient showed acceptable values for accurate assessment. For angular and linear measurements the Dahlberg’s coefficient ranged from 0.1 to 0.3° and from 0.1 to 0.2 mm respectively, while the volumetric coefficients ranged from 0.1142 to 220.7mm^2^. The percentage of the random errors in relation to the magnitude of the original measure ranged from 0.1 to 4.8%, being an acceptable, once the probabilistic limit error of 95% of confidence was established [21–23].

### Sample description

In the MAD group, the TP was on average 97.4 ± 4.8% of the maximum protrusion, ranging from 85 to 100% of the mandible’s maximum anterior displacement, this group was composed of 17 patients, being 9 males and 8 females (aged from 34 to 60), while MMA group was composed of 17 patients, 7 males and 10 females (aged from 20 to 57). In the MAD group, 15 patients showed an AHI lower than 10, after treatment indicating a successful outcome, while 3 patients demonstrated AHI lower than 36 indicating partial success. There was no statistical difference regarding the distribution by sex between the two study groups (p^b^=0.492). The mean age of the patients in the MAD group was significantly greater than in the MMA group (p^a^<0.00). Weight (p^a^=0.693), height (p^a^=0.616) and BMI (p^a^=0.223) did not differ significantly between groups. Due to the statistical difference between the age of the two groups, this variable was considered as an adjustment for the other analyzes (Table 1).

**Table 1:** Sample description

	Groups		
	MAD (n = 17)	MMA (n = 17)	p-value^a^
**Anthropometric characteristics**			
Sex (M/F)	9/8	7/10	0.492^b^
Age	47.35 ± 9.33	34.00 ± 11.20	**0.001**
Weight	70.76 ± 16.01	68.59 ± 15.89	0.693
Height	1.65 ± 0.13	1.67 ± 0.11	0.616
BMI	25.83 ± 3.32	24.38 ± 3.48	0.223

* p < 0.05, ^a^Student’s t test; ^b^Pearson’s chi-square test (n). M = male. F = female. BMI = Body mass index. MAD = mandibular advancement device. MMA = maxillomandibular advancement

### Upper airway measurements

In the MAD group, although the UA total volume and surface area in T1 was greater than in T0, no statistical difference was found in total volume (p^b^=0.142) and surface area (p^b^=0.159). In the superior oropharynx, MAD group showed a statistically significant increase in volume (p^b^=0.003) and surface area (p^b^=0.003) after MAD treatment. This group did not show statistical difference in inferior oropharynx volume (p^b^=0.247) and surface area (p^b^=0.073) between T0 and T1 (Table 2).

**Table 2:** Upper airway measurements

	Groups		Multifactorial analysis			
	MAD	MMA	p-value^a^	p-value^c^	p-value^d^	
**UA total volume**						
T0	12860.12 ± 4442.52	13485.22 ± 8376.86	0.788	0.064	0.413	
T1	14130.82 ± 4258.66	19984.25 ± 8906.67	**0.020**	0.059	0.310	
**p-value** ^**b**^	0.142	**0.003**				
**UA total area**						
T0	5380.06 ± 1245.42	5153.73 ± 1790.80	0.672	**0.036**	0.121	
T1	5685.71 ± 1297.52	6662.65 ± 1992.15	0.100	**0.016**	0.914	
**p-value** ^**b**^	0.159	**0.001**				
**Superior oropharynx volume**						
T0	7993.69 ± 2397.96	10030.88 ± 6559.56	0.238	0.249	0.726	
T1	10049.33 ± 3555.98	15248.59 ± 6946.79	**0.010**	0.903	**0.037**	
**p-value** ^**b**^	**0.003**	**0.003**				
**Superior oropharynx area**						
T0	3440.90 ± 736.27	3780.54 ± 1665.06	0.447	0.839	0.609	
T1	3836.44 ± 860.38	5100.30 ± 1821.71	**0.017**	0.992	**0.043**	
**p-value** ^**b**^	**0.003**	**0.001**				
**Inferior oropharynx volume**						
T0	4863.08 ± 2382.66	4105.69 ± 2019.74	0.325	0.422	0.210	
T1	4311.76 ± 2267.63	6183.29 ± 2338.97	**0.024**	0.396	0.148	
**p-value** ^**b**^	0.247	**< 0.001**				
**Inferior oropharynx area**						
T0	2279.50 ± 722.15	2034.82 ± 515.63	0.264	0.218	0.109	
T1	2082.07 ± 707.66	2731.81 ± 709.37	**0.012**	0.338	0.103	
**p-value** ^**b**^	0.073	**0.001**				

* p < 0.05, ^a^Student’s t test; ^b^Paired t test (mean ± SD); ^c^Multifactorial ANOVA Age factor; ^d^Multifactorial ANOVA Group factor. MAD = mandibular advancement device. MMA = maxillomandibular advancement. UA = Upper airway

In the MMA group, the UA total volume (p^b^=0.003) and surface area (p^b^=0.001) statistically increased in T1. This group also showed a significant increase in the superior oropharynx volume (p^b^=0.003) and surface area (p^b^=0.001) after the surgery. In addition, the inferior oropharynx volume (p^b^<0.001) and surface area (p^b^=0.001) were significantly greater in T1 as well (Table 2).

#### Comparison between MAD and MMA groups

No statistical difference in UA total volume was found between the groups before treatment (p^a^=0.788). However, in T1 the MMA group showed greater increase in UA total volume than the MAD group (p^a^=0.020). The age factor was considered as determinant factor for this finding, once group factor showed a p^d^=0.310 (Table 2).

The UA superior oropharynx volume in T0 did not differ between the groups (p^a^=0.238). Both groups showed a significant increase in the superior oropharynx volume after the treatments (p^b^=0.003). However, this increase in the superior volume was greater in MMA group (p^a^=0.010). This finding was not interfered by age (p^d^=0.037). The superior oropharynx area significantly increased in MAD and MMA groups. This amount of increase was higher in MMA group (p^a^=0.017) and the age was not determinant for this outcome (p^d^=0.043). (Table 2).

The UA inferior oropharynx volume (p^a^=0.325) and surface area (p^a^=0.264) did not differ at T0 comparing the groups. At the T1 CBCT, the inferior volume (p^a^=0.024) and area (p^a^=0.012) were greater in the MMA group than in the MAD group. The age was a determinant factor in inferior oropharynx volume (p^d^=0.148) and area (p^d^=0.103) (Table 2).

### Mandibular measurements

The mandibular linear anterior displacement was statistically greater (p^a^=0.010) in the MMA group (6.47 ± 4.67) than in the MAD group (2.75 ± 3.08). The mandibular linear vertical measurement showed statistical difference comparing MAD and MMA groups (p^a^ <0.001). In the MAD group (-9.29 ± 3.06), patients demonstrated a more inferior vertical position of the mandible after treatment than the MMA group patients (1.66 ± 4.32), which showed an upward vertical displacement (Table 3).

**Table 3:** Mandibular measurements

	Groups		Multifactorial analysis		
	MAD	MMA	p-value^a^	p-value^c^	p-value^d^
**Mandibular linear displacement**					
Anteroposterior	2.75 ± 3.08	6.47 ± 4.67	**0.010**	**0.026**	**0.001**
Superoinferior	-9.29 ± 3.06	1.66 ± 4.32	**< 0.001**	0.076	**< 0.001**
**Mandibular ramus angular rotation**	-3.97 ± 1.07	2.40 ± 3.43	**< 0.001**	0.024	**< 0.001**
**Mandibular anterior angular rotation**	-4.08 ± 1.30	3.41 ± 2.79	**< 0.001**	0.003	**< 0.001**

* p < 0.05, ^a^Student’s t test; ^c^Multifactorial ANOVA Age factor; ^d^Multifactorial ANOVA Group factor. MAD = mandibular advancement device. MMA = maxillomandibular advancement

The mandibular ramus angular rotation and mandibular anterior angular rotation were statistically different between the groups (p^a^<0.001). While the MAD group showed a clockwise rotation pattern (-3.97 ± 1.07 and − 4.08 ± 1.30), the MMA group demonstrated a counterclockwise (2.40 ± 3.43 and 3.41 ± 2.79). The age factor did not influence mandibular measurements outcomes (p-values^c^ equal or less than 0.001) (Table 3).

### Correlations among the measures

In the MAD group, the mandibular linear anterior displacement was correlated with superior [p = 0.002 (r=-0.697)] and inferior [p = 0.004 (r = 0.658)] oropharynx volume, suggesting that greater amounts of mandibular advancement are correlated to a decrease in the superior oropharynx and an increase in the inferior oropharynx. Mandibular anterior angular rotation was correlated with mandibular linear displacement in an inferior direction [p = 0.020 (r = 0.557)], clockwise mandibular rotation (Table 4).

**Table 4:** Correlation between mandibular linear anterior rotation and groups variables

	Mandibular linear displacement Anteroposterior	Mandibular linear displacement Superoinferior
**MAD**		
ΔSuperior oropharynx	p = 0.002 (r=-0.697)*	p = 0.186 (r = 0.337)
ΔInferior oropharynx	p = 0.004 (r = 0.658)*	p = 0.485 (r=-0.182)
Mandibular ramus angular rotation	p = 0.211 (r = 0.320)	p = 0.963 (r=-0.012)
Mandibular anterior angular rotation	p = 0.103 (r = 0.409)	p = 0.020 (r = 0.557)*
**MMA**		
ΔSuperior oropharynx	p = 0.029 (r=-0.530)*	p = 0.047 (r = 0.488)*
ΔInferior oropharynx	p = 0.208 (r = 0.322)	p = 0.092 (r=-0.422)
Mandibular ramus angular rotation	p = 0.001 (r = 0.743)*	p = 0.397 (r = 0.220)
Mandibular anterior angular rotation	p = 0.000 (r = 0.785)*	p = 0.000 (r = 0.753)*

*p < 0.05, Pearson correlation. MAD = mandibular advancement device. MMA = maxillomandibular advancement

In the MMA group, the superior oropharynx volume was correlated to mandibular mandibular anteroposterior [p = 0.029 (r=-0.530)] and vertical displacement [p = 0.047 (r = 0.488)]. This analysis suggests that greater amounts of mandibular advancement may lead to a lowest gain in the superior oropharynx volume, while a great mandibular superior displacement is correlated with better improvements in this UA region. Mandibular ramus angular rotation was correlated with the mandibular anteroposterior linear displacement [p = 0.001 (r = 0.743)]. Mandibular anterior angular rotation was correlated with both mandibular linear displacements, anteroposterior [p = 0.000 (r = 0.785]) and superoinferior [p = 0.000 (r = 0.753)]. This outcome may imply that greater amounts of mandibular linear displacements are correlated to a counterclockwise rotation pattern (Table 4).

## Discussion

The present study evaluated and compared upper airway volume and surface area, as well as mandibular rotation in patients undergoing either intraoral treatment with MAD and MMA. The assessments of these therapies’ effects are important for treatment planning when the objectives include increasing the airway dimensions, preventing or treating sleep breathing disorders, such as obstructive sleep apnea.

The MAD group did not present a significant increase of the UA total volume and area at T1. However, the superior oropharynx volume and surface area demonstrated a statistically significant increase. These findings were similar to the outcomes of Barbero et al.[24], which demonstrated that the superior portions of UA are mostly affected by MAD. These authors reported that the velopharynx was the region with largest volume in all studied appliance positions [24].

The UA total volume and surface area in the MMA group significantly increased 1 year after surgery. This increase was significant in both the superior and inferior oropharynx regions. These findings agree with Marcussen et al.[25], who demonstrated statistical increases in velopharynx and oropharynx after MMA for Class II treatment. Gurani at al.[26] also identified a statistically relevant UA volume increase of 26% immediately after the MMA. However, the authors identified a loss in volume of 20% after 2-years. The loss in volume gain was also reported in a study that identified an increase in total volume and area, as well as in nasopharynx, oropharynx and hypopharynx. Nevertheless, the reported losses in volume and area started at 1 year after surgery [27–29].

The total, superior, inferior volume and area of the oropharynx were statistically greater at T1 in the MMA group than in the MAD group. This finding may be explained by the mandible movement pattern from each group. In the MAD group the amount of mandible advancement achieved was significantly smaller, while the vertical component was greater in the MAD group compared with the MMA group. The MAD group showed clockwise mandibular rotation and the MMA group demonstrated a counterclockwise mandibular rotation 1 year after surgery. It has been reported that MAD treatment may increase vertical dimensions and that the amount of resultant mandibular protrusion is reduced 0.3 mm for each 1 mm of vertical displacement [18, 30]. This change in the vertical dimension occurs due to the design of the oral appliance may interfere in the amount of mandibular advancement and rotation. Such clockwise rotational pattern with the oral device was associated with greater gain in volume and area in the superior portion of the UA but not in the inferior oropharynx region. The MAD used in the present study was designed with a vertical height of 7 mm and in the therapeutic position, the patients showed an average vertical inferior displacement of 9.29 mm. This displacement was directly correlated with the mandibular clockwise rotation pattern. This finding hightlight the importance of selecting a mandibular advancement device with a minimal vertical dimension.

In surgical patients, the counterclockwise rotation pattern leads to gains in UA volume and airway area in general, both in superior and inferior oropharynx portions [6]. The surgery group also demonstrated that the mandibular advancement and vertical superior displacement were directly correlated with greater dimensions in superior oropharynx and most of the counterclockwise rotation variables. These outcomes are in agreement with Marcussen et al.[25] who identified that counterclockwise rotation were correlated to velopharynx and glossopharynx volume improvement. In both groups, greater amounts of the mandibular anterior displacement (measured by the distance between B point in T0 and B point in T1) were correlated with a reduced gain in the superior oropharynx volume. This statistically significant finding indicates that the amount of mandibular advancement for adequate UA patency is limited, and the improvement in the superior oropharynx volume may be obtained by balanced amount of advancement rather than larger anterior displacements. The use of point B for evaluation of the mandibular anteroposterior and vertical position was proposed by Steiner, and since then has been widely used in orthodontics. As the Menton, Pogonion and Gnathion points are located in the mandibular symphysis, these landmarks are more often affected by changes in the position and shape of the chin [31, 32].

The UA may be improved by both therapies at different levels, ways and quantities. The MMA treatment improved UA dimensions in all UA regions by a counterclockwise mandibular rotation, leading to a gain of great amount of volume and surface area. On the other hand, the MAD group had mainly increase in the superior portion of the UA by a clockwise mandibular movement with lesser volume and area improvements than the comparison group. The MMA seems to be the most effective option to increase the UA. Nonetheless, MMA is also a more invasive treatment and studies have demonstrated loss in volume gain in long-term evaluations [26–29], while MAD is a more conservative therapy that is efficient for the superior UA portion. In both groups, no patient reported TMD symptoms with treatment. Despite the fact that temporomandibular disorders have been described as a possible adverse effect of MAD treatment, this intraoral appliance therapy is not irreversible. Once the patient demonstrates any collateral effect, the treatment may be interrupted before severe consequences, being an extremely conservative therapy option.

A validated image analysis protocol is important for quantitative assessments of UA 3D morphology and treatment changes in this anatomic region. Considering the patient position during the image acquisition, it is reported the due the gravitational forces and the flexibility of the airway soft tissues, the upright and supine positions may influence the UA dimension. To address this fact the patients from both samples performed the CBCT scan at the same upright position [33]. The mandibular and upper airway measurements were made with standardized head orientation and registration between the scans obtained before and after treatment. Importantly, the voxel based registration uses the raw information of the image, comparing patient specific grey scale intensity of the voxels between the T0 and T1 scans of the same patient. Due to its better accuracy and reduced possibility of errors, the voxel based registration was performed in the present study [20, 34].

The 3D Slicer tools and ITK-SNAP semi-automatic segmentation were used in the present study [20]. Both software demonstrated accuracy and precision comparable to Dolphin imaging analyzes [35–37]. Moreover, the methodology used to standardize the CBCT measurement protocol followed the suggestions of a recent systematic review that, based on the studies with a low risk of bias, established steps for precise evaluation of the UA with CBCT [38].

A limitation of this study was the age difference between the groups. However, the statistical analysis found that the age factor interfered with only three of the variables measured. Moreover, it was not possible to evaluate the sleep disorders in this study due to the fact that the surgical group did not perform PGS exams, being unfeasible to extend the analysis beyond the anatomic factors. For this reason, the present study aimed only to the evaluated the anatomic changes, such as the UA changes and mandibular rotation with both treatments. Another limitation is the retrospective design, and due to the relevance of the findings prospective case-control studies are encouraged. Up to date, there is a gap in the literature in comparing upper airway patency and mandibular movement between patients treated with MAD and MMA. Therefore, future longitudinal investigations on how the observed morphological and positional changes affect airway patency are still needed.

Importantly, these findings highlight the importance of considering a balanced amount of advancement to a success outcome in both therapies. Moreover, this study shows that the knowledge of the effects of these treatments on the UA dimensions and mandibular rotation are essential to guide the clinician’s decision regarding the treatment of choice to increase the UA, analyzing the individual needs and cost benefit, towards successful outcomes in the treatment or prevention of diseases related to UA patency.

## Conclusions

- MAD increased only the superior oropharynx volume and surface area.
- MMA increased total upper airway, as well as superior and inferior oropharynx volume and surface area.
- MMA treatment achieved greater volume and area in all UA regions compared to MAD treatment.
- The UA dimensions improvement occurred in both therapies by different mandibular movements.
- In both MAD and MMA groups, greater amounts of the mandibular anterior displacement were correlated with a reduced gain in the superior oropharynx volume, highlighting the importance of planning the amount of advancement for each patient.
- The clinician’s decision regarding the best treatment should be guided by the patient’s needs, amount of necessary advancement, costs, and expected benefits.

## Electronic supplementary material

Below is the link to the electronic supplementary material.

Supplementary Material 1

## Data Availability

The datasets used and/or analyzed during the current study are available from the corresponding author on reasonable request.
