# Supplementary material for: Three-dimensional comparison between the effects of mandibular advancement device and maxillomandibular advancement surgery on upper airway
Source: BMC Oral Health. 2023 Jun 30;23:436. doi: 10.1186/s12903-023-03125-5 (PMC10314553; doi:10.1186/s12903-023-03125-5)
Supplement: Supplementary file 1 — Supplementary Material 1 [file 12903_2023_3125_MOESM1_ESM.docx]

Table S1: Landmarks list for mandibular and upper airway measurements.

| Abbreviation | 3D landmarks location: | Lateral | Axial | Anteroposterior |
| --- | --- | --- | --- | --- |
| B | B point | Located in the largest concavity of the anterior portion of the mental symphysis | Deepest point of the mental symphysis | Deepest point along anterior concavity of maxilla |
| RCo | Right Condylion | Most superior point of right condyle contour | Most superior and central point of right condyle contour | Most superior point of right condyle contour |
| RGo | Right Gonion | Most inferior and posterior point of right mandibular angle | Most inferior, posterior and central point of right mandibular angle | Most inferior, posterior and central point of right mandibular angle |
| LCo | Left Condylion | Most superior point of left condyle contour | Most superior and central point of left condyle contour | Most superior point of left condyle contour |
| LGo | Left Gonion | Most inferior and posterior point of left mandibular angle | Most inferior, posterior and central point of left mandibular angle | Most inferior, posterior and central point of left mandibular angle |
| C2I | Second Vertebra point | Most inferior and anterior point of the second cervical vertebra | Most inferior and anterior point of the second cervical vertebra | Most inferior and anterior point of the second cervical vertebra |
| C4S | Fourth Vertebra Point | Most superior and anterior point of the fourth cervical vertebra | Most superior and anterior point of the fourth cervical vertebra | Most superior and anterior point of the fourth cervical vertebra |
| Ba | Basion | Most inferior point of foramen magnum anterior margin | Most anterior and central point of foramen magnum | Most inferior and central point of foramen magnum |
| PNS | Posterior Nasal Spine | Most posterior point of hard palate | Most posterior and central point of hard palate | Most posterior and central point of hard palate |
